# Population Pharmacokinetic Model and Meta-analysis of Outcomes of Amphotericin B Deoxycholate Use in Adults with Cryptococcal Meningitis

**DOI:** 10.1128/AAC.02526-17

**Published:** 2018-06-26

**Authors:** Katharine E. Stott, Justin Beardsley, Sarah Whalley, Freddie Mukasa Kibengo, Nguyen Thi Hoang Mai, Ruwanthi Kolamunnage-Dona, William Hope, Jeremy Day

**Affiliations:** a Antimicrobial Pharmacodynamics and Therapeutics Laboratory, Department of Molecular and Clinical Pharmacology, Institute of Translational Medicine, University of Liverpool, Liverpool, United Kingdom; b Malawi-Liverpool-Wellcome Trust Clinical Research Programme, Blantyre, Malawi; c Oxford University Clinical Research Unit, Ho Chi Minh City, Vietnam; d MRC/UVRI Uganda Research Unit on AIDS, Entebbe, Uganda; e Department of Biostatistics, Institute of Translational Medicine, University of Liverpool, Liverpool, United Kingdom; f Hospital for Tropical Diseases, Ho Chi Minh City, Vietnam; g Centre for Tropical Medicine and Global Health, Nuffield Department of Medicine, University of Oxford, Oxford, United Kingdom

**Keywords:** cryptococcal meningitis, pharmacokinetics, pharmacodynamics, amphotericin B deoxycholate, meta-analysis, population pharmacokinetics

## Abstract

There is a limited understanding of the population pharmacokinetics (PK) and pharmacodynamics (PD) of amphotericin B deoxycholate (DAmB) for cryptococcal meningitis. A PK study was conducted in *n* = 42 patients receiving DAmB (1 mg/kg of body weight every 24 h [q24h]). A 2-compartment PK model was developed. Patient weight influenced clearance and volume in the final structural model. Monte Carlo simulations estimated drug exposure associated with various DAmB dosages. A search was conducted for trials reporting outcomes of treatment of cryptococcal meningitis patients with DAmB monotherapy, and a meta-analysis was performed. The PK parameter means (standard deviations) were as follows: clearance, 0.03 (0.01) × weight + 0.67 (0.01) liters/h; volume, 0.82 (0.80) × weight + 1.76 (1.29) liters; first-order rate constant from central compartment to peripheral compartment, 5.36 (6.67) h^−1^; first-order rate constant from peripheral compartment to central compartment, 9.92 (12.27) h^−1^. The meta-analysis suggested that the DAmB dosage explained most of the heterogeneity in cerebrospinal fluid (CSF) sterility outcomes but not in mortality outcomes. Simulations of values corresponding to the area under concentration-time curve from h 144 to h 168 (AUC_144–168_) resulted in median (interquartile range) values of 5.83 mg · h/liter (4.66 to 8.55), 10.16 mg · h/liter (8.07 to 14.55), and 14.51 mg · h/liter (11.48 to 20.42) with dosages of 0.4, 0.7, and 1.0 mg/kg q24h, respectively. DAmB PK is described adequately by a linear model that incorporates weight with clearance and volume. Interpatient PK variability is modest and unlikely to be responsible for variability in clinical outcomes. There is discordance between the impact that drug exposure has on CSF sterility and its impact on mortality outcomes, which may be due to cerebral pathology not reflected in CSF fungal burden, in addition to clinical variables.

## INTRODUCTION

Cryptococcal meningitis is a leading infectious cause of morbidity and mortality worldwide, with approximately 223,100 incident cases and 181,100 deaths annually (1). The 10-week mortality rate for patients receiving the current standard of care is 24% to 31% (2–5). There have been no new antifungal agents developed for use in low-to-middle-income countries in the last 3 decades. Given the paucity of new agents, one important strategy for improving clinical outcomes is a better understanding and use of currently available compounds.

Amphotericin B (AmB) is a polyene antifungal agent with broad-spectrum activity against yeasts and molds, as well as against some parasites. AmB was initially isolated from a streptomycete and was originally described in 1955 (6). AmB was the first therapeutic option for treatment of lethal invasive fungal diseases such as cryptococcal meningitis (7, 8). Amphotericin B deoxycholate (DAmB) is the most potent formulation of AmB on a milligram-to-milligram basis (9, 10) and is a mainstay for the treatment of cryptococcal meningitis.

Clinical studies have progressively examined escalating dosages of 0.4 mg/kg of body weight every 24 h (q24h) (11, 12), 0.7 mg/kg q24h (13–15), and 1.0 mg/kg q24h (5) of DAmB for cryptococcal meningitis. The primary motivation of these studies was identification of the dosage that induces maximal antifungal activity. A regimen of 1.0 mg/kg q24h in combination with 5FC (flucytosine) for 1 week is currently recommended for induction therapy (16). High DAmB dosages are associated with increased rates of cerebrospinal fluid (CSF) sterilization (2) and improved mortality (4, 5, 17). However, the broad clinical utility of DAmB is compromised by dose-limiting toxicities that include infusional reactions, phlebitis, nephrotoxicity, and anemia (18, 19). A detailed understanding of the therapeutic index for each DAmB dosage level is lacking.

Here, we describe the development of a population pharmacokinetic model of DAmB. In addition, a meta-analysis of clinical trials of DAmB monotherapy was performed to estimate the contribution of various DAmB dosages to the observed heterogeneity in study outcomes. Finally, Monte Carlo simulations were performed to estimate the mean, median, and dispersion values corresponding to drug exposures that are associated with microbiological and clinical outcomes of DAmB monotherapy.

## RESULTS

### Demographics.

A total of 42 patients (22 from Vietnam and 20 from Uganda) were recruited over an 11-month period between January and November 2016. A total of 22 (52%) of the patients were female. The overall median (range) age was 33 years (20 to 73 years), the overall median weight was 48 kg (32 to 68 kg), the overall median body mass index was 18 kg/m^2^ (12 to 25 kg/m^2^), the overall median level of creatinine at enrollment was 69 μmol/liter (37 to 167 μmol/liter), and the overall median estimated glomerular filtration rate (determined using the Cockcroft-Gault equation) was 76.7 ml/min/1.73 m^2^ (35.4 to 146.7 ml/min/1.73 m^2^). The demographic data are shown by ethnicity and overall in Table 1. There were no statistically significant differences between ethnic groups in any demographic variable.

**TABLE 1:** Patient demographics

Demographic or clinical characteristic^*a*^	Value(s)			*P* value for difference between Vietnam and Uganda data
	Vietnam	Uganda	Combined	
Sex^*b*^ (no. of males:no. of females)	12:10	8:12	20:22	
Age (yrs)^*c*^				
Mean	38	33	36	0.75^*g*^
Median	33	33	33	
Range	20–73	24–50	20–73	
Weight (kg)^*d*^				
Mean	47	49	48	0.21^*g*^
Median	46	49	48	
Range	32–68	35–60	32–68	
BMI (kg/m^2^)^*e*^				
Mean	18	18	18	0.73^*h*^
Median	18	19	18	
Range	12–25	15–22	12–25	
Creatinine (μmol/liter)^*b*^				
Mean	71	81	75	0.06^*g*^
Median	62	79	69	
Range	37–167	43–145	37–167	
eGFR (ml/min/1.73 m^2^)^*f*^				
Mean	90.6	79.3	84.7	0.19^*g*^
Median	89.8	73.5	76.7	
Range	35.4–136.1	49.8–146.7	35.4–146.7	

a BMI, body mass index; eGFR, estimated glomerular filtration rate (by Cockcroft-Gault equation).

b *n* = 42.

c *n* = 28.

d *n* = 39.

e *n* = 33.

f *n* = 26.

g Mann-Whitney test of significance.

h Unpaired *t* test of significance.

### Pharmacokinetic data.

**FIG 1:**
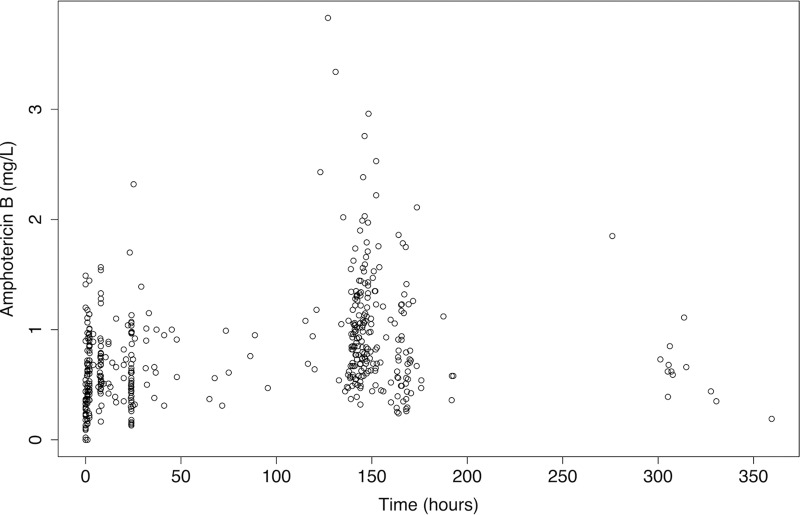
Amphotericin B serum concentrations in 42 patients. Patients received 1.0 mg/kg of amphotericin B deoxycholate (DAmB), infused over 5 to 6 h.

The final data set included 282 of 312 total observations from the Vietnamese cohort and 197 of 241 total observations from the Ugandan cohort (mean, 11.4 samples per patient; range, 6 to 18). In total, 74 plasma samples were excluded because of absent information regarding the time that the pharmacokinetic (PK) samples were drawn. Figure 1 shows the raw plasma concentration-time profiles from study participants.

### Population pharmacokinetic models.

Initial exploration of structural models revealed that a two-compartment model fitted the data better than a three-compartment model. Specifically, the three-compartment structural model resulted in a more negative log likelihood value (−55.5 versus −42.8) and a higher Akaike information criterion (AIC) value (127.3 versus 101.9). Accordingly, subsequent model development was based on a two-compartment base model.

**FIG 2:**
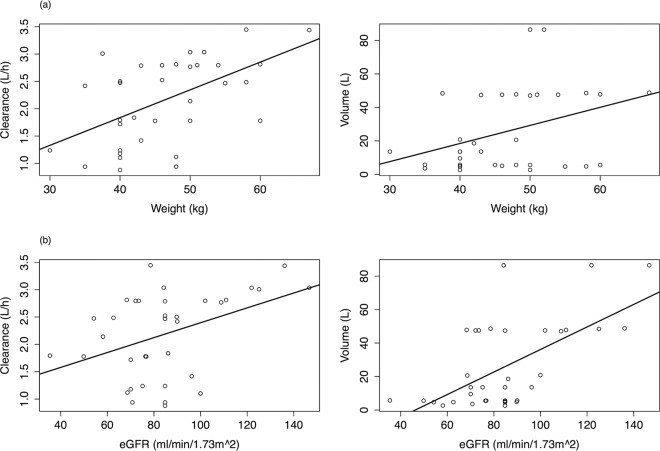
Linear regression of the relationship between (a) patient weight and (b) estimated glomerular filtration rate and Bayesian posterior estimates for clearance and volume of distribution. Circles are Bayesian estimates from each patient. Solid line: linear regression. (a) (Left panel) *R*^2^ = 0.32. Clearance = 0.05 · weight − 0.2. (Right panel) *R*^2^ = 0.12. Volume = 1.08 · weight − 24.8. (b) (Left panel) *R*^2^ = 0.17. Clearance = 0.01 · eGFR + 1.03. (Right panel) *R*^2^ = 0.36. Volume = 0.67 · eGFR − 31.13.

Model 1 was a standard two-compartment model without inclusion of covariates. Linear regressions of the Bayesian estimates of clearance and volume (derived from the mean population PK parameter values from model 1) with weight and estimated glomerular filtration rate (eGFR) as covariates are presented in Fig. 2a and b, respectively. A relationship was apparent between patient weight and both estimated clearance (slope, 0.05; 95% confidence interval [CI] for estimate of slope, 0.02 to 0.08; *P* = 0.002) and estimated volume of the central compartment (slope, 1.08; 95% CI, 0.05 to 2.11; *P* < 0.001). Similarly, linear regression data showed a positive relationship between eGFR and estimated clearance (slope of linear regression, 0.01; 95% CI for the slope, 0 to 0.02; *P* < 0.05) and volume (slope, 0.67; 95% CI, 0.36 to 0.98; *P* < 0.001). These covariates were incorporated into the structural model as follows: model 2 incorporated weight as a covariate with a linear term for clearance; model 3 incorporated weight as a covariate with a nonlinear term for clearance; and model 4 incorporated both weight and baseline renal function as covariates in the structural model, with linear clearance. Population PK parameter estimates for all 4 models are shown in Table 2. There were no statistically significant differences in the estimated clearance and volume data from the standard model (model 1) according to ethnicity. The mean (95% CI) values for clearance were 2.03 liters/h (1.69 to 2.38) and 2.24 liters/h (1.91 to 2.56) for Vietnamese and Ugandan patients, respectively (*P* value, 0.37). The mean (95% CI) volumes were 33.55 liters (17.96 to 49.13) and 63.93 liters (40.98 to 86.88) for Vietnamese and Ugandan patients, respectively (*P* value, 0.09).

**TABLE 2:** Parameter estimates for the initial and modified two-compartment pharmacokinetic models

Parameter^*a*^	Value		
	Mean	Median	SD
Model 1			
SCL (liters/h)	2.19	2.46	0.77
Vc (liters)	27.77	13.88	28.06
K12 (h^−1^)	3.84	2.16	6.57
K21 (h^−1^)	1.14	0.32	3.06
Model 2			
SCL_slope_ (liters/h/kg)	0.03	0.03	0.01
SCL_intercept_ (liters/h)	0.67	0.57	0.01
Vc_slope_ (liters/kg)	0.82	0.36	0.80
Vc_intercept_ (liters)	1.76	1.99	1.29
K12 (h^−1^)	5.36	3.83	6.76
K21 (h^−1^)	9.92	0.46	12.27
Model 3			
SCL (liters/h/kg)	0.12	0.12	0.04
Vc (liters/kg)	1.40	0.51	1.75
K12 (h^−1^)	1.69	0.50	4.09
K21 (h^−1^)	8.31	0.27	12.32
Model 4			
SCL_slope_ (liters/h/kg)	0.01	0.01	0.01
SCL_intercept_ (liters/h)	1.50	1.31	0.74
Vc_slope_ (liters/kg)	1.26	0.52	1.39
Vc_intercept_ (liters)	1.64	0.01	2.96
K12 (h^−1^)	3.86	0.73	7.74
K21 (h^−1^)	11.24	0.40	13.44

a SCL, first-order clearance of drug (in liters per hour) from the central compartment; Vc, volume of distribution in the central compartment; K12, first-order rate constant from the central compartment to the peripheral compartment; K21, first-order rate constant from the peripheral compartment to the central compartment.

The fit of the model to the data was acceptable for all 4 two-compartment models. The model diagnostics are presented in Table 3. The values corresponding to the coefficient of determination of a linear regression of observed-versus-predicted plots after the Bayesian step were 0.72, 0.74, 0.69, and 0.73 for models 1, 2, 3, and 4, respectively. The values for intercept and slope approximated 0 and 1, respectively, for each regression (Table 3). The mean parameter values predicted the observed values better than the medians. The measures of population bias and imprecision were comparable between the models, with bias values of −0.85, −0.34, −0.23, and −0.43 and imprecision values of 3.13, 2.97, 2.16, and 3.29 for models 1, 2, 3, and 4, respectively. The more positive log likelihood value and lower Akaike information criterion (AIC) value for model 2 suggested that the inclusion of weight as a covariate explained a portion of the observed variance.

**TABLE 3:** Evaluation of the predictive performance of the initial model and final model^*a*^

Model	Log likelihood	No. of cycles to convergence	AIC	Population bias	Population imprecision	Linear regression of observed-predicted values for each patient		
						*R*^2^^*b*^	Intercept	Slope
Model 1	−56.3	1,137	124.8	−0.85	3.13	0.72	0.08	0.97
Model 2	−42.8	1,251	101.9	−0.34	2.97	0.74	0.01	1.01
Model 3	−102.7	577	221.7	−0.23	2.16	0.69	0.00	1.04
Model 4	−43.1	1,704	102.7	−0.43	3.29	0.73	0.01	1.02

a Model 2 included a linear function to scale DAmB clearance to patient weight. Model 3 included a nonlinear function to scale DAmB clearance to patient weight. Model 4 included a function to scale DAmB clearance to patient weight and eGFR.

b *R*^2^ values are expressed relative to the regression line fitted for the observed versus predicted values after the Bayesian step.

**FIG 3:**
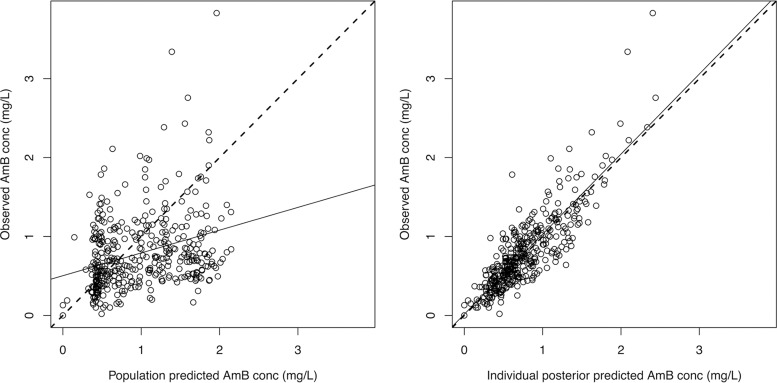
Scatter plots showing observed versus predicted values for the chosen population pharmacokinetic model after the Bayesian step (model 2). (Left panel) *R*^2^ = 0.17. Intercept = 0.18 (95% CI, 0.03 to 0.32). Slope = 0.89 (95% CI, 0.70 to 1.09). (Right panel) *R*^2^= 0.74. Intercept = 0.01 (95% CI, −0.04 to 0.07). Slope = 1.01 (95% CI, 0.95 to 1.07). Circles, dashed lines, and solid lines represent individual observed-predicted data points, the line of identity, and the linear regression of observed and predicted values, respectively. All observed and predicted amphotericin B concentrations (conc) are indicated in milligrams per liter. AmB, amphotericin B; CI, confidence interval.

**FIG 4:**
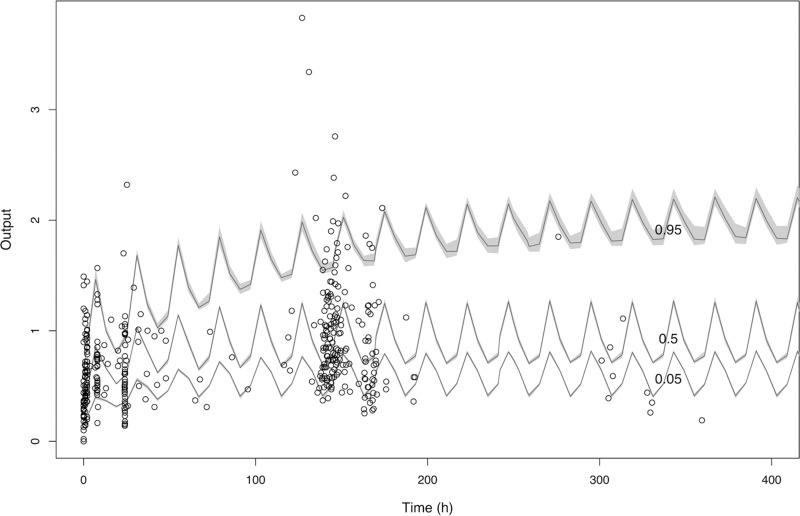
Visual predictive check of the final model. The black circles indicate observed DAmB concentrations. The continuous lines represent the 5th, 50th, and 95th percentiles of DAmB concentrations for 1,000 simulated patients. In total, 83.4% of observed DAmB concentrations fall within the 5th and 95th percentiles estimated by the final model, indicating adequate model fit.

The model that incorporated an exponential term for clearance (model 3) decreased the log likelihood value and increased the AIC value (Table 3). The inclusion of eGFR in model 4 failed to increase the log likelihood value or reduce the AIC value further. In addition, there was no statistically significant difference between model 1 and either model 3 or model 4; the latter models were therefore rejected. Model 2 was chosen as the final model. Observed-versus-predicted plots for the population and Bayesian posterior values in the final model are shown in Fig. 3. Figure 4 shows a visual predictive check (VPC) of the final model.

### Meta-analysis of clinical outcome data.

**FIG 5:**
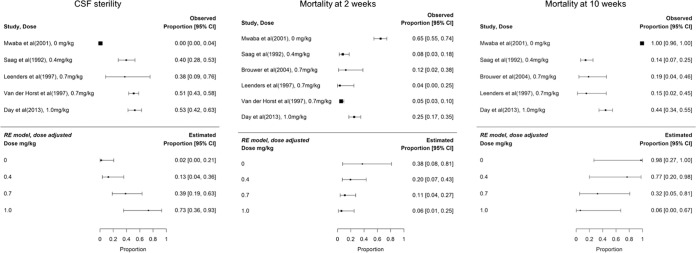
Meta-analysis of clinical trials of DAmB monotherapy, showing dose-adjusted effects on CSF sterility (left panel), mortality at 2 weeks (middle panel), and mortality at 10 weeks (right panel). (Left panel) Tau value for unadjusted model: 4.22. Tau value for dose-adjusted model: 0.98. Dose adjustment accounts for (4.22 − 0.98)/4.22 = 77% of the heterogeneity in clinical outcomes. *P* value for dose adjustment, 0.007. (Middle panel) Tau value for unadjusted model: 1.90. Tau value for dose-adjusted model: 1.28. Dose adjustment accounts for (1.90 − 1.28)/1.90 = 33% of the heterogeneity in clinical outcomes. *P* value for dose adjustment, 0.14. (Right panel) Tau value for unadjusted model: 9.0. Tau value for dose-adjusted model: 4.93. Dose adjustment accounts for (9.00 − 4.93)/9.00 = 45% of heterogeneity in clinical outcomes. *P* value for dose adjustment, 0.07. RE, random effects. Day et al., reference 5; Saag et al., reference 11; Leenders et al., reference 13; van der Horst et al., reference 14; Brouwer et al., reference 15; Mwaba et al., reference 20.

Five clinical trials that included a DAmB monotherapy arm were identified. There was one trial in which 63 patients received 0.4 mg/kg of body weight q24h (11), 3 trials in which a combined total of 208 patients received 0.7 mg/kg q24h (13–15), and 1 trial in which 99 patients received 1.0 mg/kg q24h (5). An additional study that reported clinical outcomes in untreated cryptococcal meningitis patients was also included. The baseline variables and clinical outcomes of these study arms are summarized in Table 4. Due to the small number of studies, we were unable to adjust for baseline variables that may have had an impact on outcome measures (e.g., age, CD4 cell count, baseline level of consciousness, baseline fungal burden, and baseline cryptococcal antigenemia). The forest plots of the dose-adjusted random-effects model are shown in Fig. 5. The model suggests that dose adjustment accounted for 77% of the heterogeneity in CSF sterility (*P* = 0.007) but that it did not have a significant impact on the heterogeneity in either the 2-week mortality outcomes (33%, *P* = 0.14) or the 10-week mortality outcomes (45%, *P* = 0.07).

**TABLE 4:** Clinical outcomes from trial data of DAmB monotherapy, by dosing regimen^*a*^

DAmB regimen (mg/kg q24h)	Location(s)	No. of patients	Median age (yrs)	Median CD4 cell count per mm^3^	No. of patients with reduced LOC at baseline/total no. of patients (%)	Median baseline fungal burden, log_10_ CFU/ml (range)	Baseline CSF CrAg titer, median	Documented CSF sterility/total no. of patients (%)	No. of patients with mortality at 2 wks/total no. of patients (%)	No. of patients with mortality at 10 wks/total no. of patients (%)	No. of patients with indicated grade of anemia/total no. of patients (%)	No. of patients with hypokalemia/total no. of patients (%)	Reference
No treatment	Zambia	100	32	NR	NR	NR	NR	*0/100* (presumed)	65/100 (65)	100/100 (100)	NR	NR	20
0.4	United States	63	37	NR	16/63 (25)	NR; *∼4.2*	1:512^*c*^	25/63 (40)	5/63 (8)	NR; 9/63 at 12 wks (14)	NR	NR	11
0.7	Thailand	16	34	9	1/16 (6)	5.63 (5.19–5.97)	1:512	NR	2/16 (13)	3/16 (19)	NR	NR	15
0.7	Australia/The Netherlands	13	41	35	2/13 (15)	NR; *∼3.9*	1:256	3/8 (37)	0/13 (0)	2/13 (15)	−20 (−45 to 5)^*b*^ at 10 wks; fall in Hb > 2 g/dl, 2/13 (15)	4/13 (31); <3 mEq/liter	13
0.7	United States	179	37	18	18/179 (10)	NR; *∼4.8*	1:1,024	91/179 (51)	11/202 (5)	NR	NR in comparable manner	NR in comparable manner	14
1.0	Vietnam	99	28	18	31/97 (32)	5.91 (5.49–6.48)	NR; *∼1:4,096*	52/99 (53)	25/99 (25)	44/99 (44)	All anemia, 62/99 (63); grade 3–4 anemia (<8 g/dl), 46/99 (46)	All, 54/99 (55); grade 3–4 (<2.5 mmol/liter), 20/99 (20)	5

a LOC, level of consciousness; NR, not reported; CSF, cerebrospinal fluid; CrAg, cryptococcal antigen; Hb, hemoglobin; mEq, milliequivalents. Italic text indicates values that were extrapolated from available data.

b % decrease in hemoglobin, median (range).

c Reported in reference 14.

### Monte Carlo simulations.

**FIG 6:**
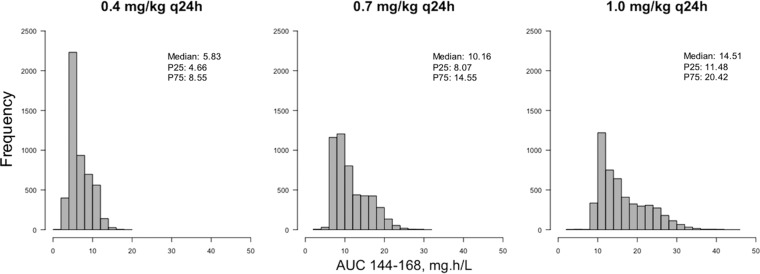
AUC distributions based on Monte Carlo simulations. Simulated dosing regimens are 0.4, 0.7, and 1.0 mg/kg q24h. Medians, 25th percentiles (P25), and 75th percentiles (P75) are displayed on each histogram.

Monte Carlo simulations (*n* = 5,000) were performed using the final population PK model. This enabled exploration of the consequences of the population PK variability, quantified in the final model, on plasma DAmB concentrations in a simulated population receiving the dosage regimens for which clinical trial outcome data were available. The median (interquartile range) area under the concentration-time curve from h 144 to h 168 (AUC_144–168_) was 5.83 mg/liter · h (4.66 to 8.55 mg/liter · h) for patients receiving DAmB 0.4 mg/kg q24h, 10.16 mg/liter · h (8.07 to 14.55 mg/liter · h) for 0.7 mg/kg q24h, and 14.51 mg/liter · h (11.48 to 20.42 mg/liter · h) for 1.0 mg/kg q24h. The AUC_144–168_ distributions from the simulations are shown in Fig. 6.

## DISCUSSION

We conducted a PK study in HIV-positive adults with cryptococcal meningitis in regions of high disease burden and developed a population PK model that enabled the extent of interpatient variability to be quantified. We described the PK of DAmB using a 2-compartment PK model with intravenous (i.v.) infusion and first-order clearance of drug from the central compartment. Simulated AUC values revealed relatively modest PK variability, suggesting that the frequently poor clinical outcomes are not the result of significant PK variability. The relationship between weight and drug clearance suggests that weight accounts for a portion of the observed variance. Dosage adjustment on the basis of weight is necessary to ensure that lighter patients are not overdosed and heavier patients are not underdosed. However, the lack of impact of either eGFR or ethnicity on the PK data suggests that dosage adjustment for these variables is not necessary to achieve comparable levels of drug exposure across patient populations.

The model-simulated median AUC value of 10.17 mg · h/liter following a regimen of 0.7 mg/kg of body weight q24h is consistent with AUC values estimated using noncompartmental techniques. For example, Bekersky et al. calculated an AUC_0–24_ value of 13.9 ± 2 mg · h/liter after administration of 0.6 mg/kg i.v. in healthy volunteers (21). However, the simulations performed following administration of 1 mg/kg resulted in a median AUC value of 14.51 mg · h/liter, which is considerably lower than that derived from a noncompartmental analysis (NCA) conducted by Ayestarán et al. for the same dose administered to neutropenic patients (28.98 ± 15.46 mg · h/liter) (22). The reason for this is not immediately clear but may relate to physiological differences between these two critically unwell patient cohorts (23).

Our meta-analysis of clinical outcomes of studies of DAmB monotherapy is limited by the fact that the included studies recorded CSF sterility at diverse time points ranging from 2 weeks (14) to 10 weeks (11). Nevertheless, the meta-analysis suggests that the dosage of DAmB has a significant impact on the proportion of patients with sterile CSF and that achieving CSF sterility is dose dependent up to 1 mg/kg q24h. However, the DAmB data did not have a dose-dependent relationship with mortality rates at either 2 or 10 weeks.

The potential reasons that DAmB dosage had a positive impact on CSF sterilization but not on mortality are as follows. First, AmB toxicity may contribute to mortality (18, 19). Nephrotoxicity is dose dependent and likely multifactorial. It is associated with a 4.5× increase in the odds of mortality from cryptococcal meningitis at 10 weeks (18). Free drug interacts with the distal tubules of the nephron causing increased monovalent ion delivery, with consequent afferent arteriolar constriction (24). Direct tubular toxicity results in hypokalemia and hypomagnesemia, leading to cardiotoxicity (24, 25). Conversely, rapid infusion of AmB can result in an extracellular shift of potassium, causing hyperkalemia and cardiac dysrhythmias (26). Anemia occurs in up to 75% of patients treated with DAmB as a result of direct suppression of erythropoiesis (24). Severe anemia more than doubles the odds of 10-week mortality from cryptococcal meningitis (18). Second, mortality may be driven by factors not directly resulting from either disease or treatment. For example, nosocomial bacteremia may occur in up to 15% to 18% of patients hospitalized for cryptococcal meningitis (27). Third, fungal burden—and therefore, conceivably, time to CSF sterility—is just one of multiple clinical variables associated with mortality in cryptococcal meningitis. Older age, altered mental status, low body weight, high peripheral white blood cell count, and anemia are independently associated with mortality at either 2 or 10 weeks (4). Immune reconstitution inflammatory syndrome (IRIS) remains a significant cause of mortality, occurring in 3% to 49% of cryptococcal meningitis patients who survive to initiation of antiretroviral treatment and carrying a mortality rate of up to 36% (17, 28). Raised intracranial pressure is an additional factor associated with mortality, and treatment incorporating at least 1 therapeutic lumbar puncture imparts a relative survival advantage of 69% in the first 10 days of treatment (29). Finally, the trial cohorts included in the meta-analysis were from diverse sites in Africa, Asia, Europe, and the United States. Factors such as health-seeking behavior and nutritional status may have influenced the mortality outcomes. Our meta-analysis did not include any baseline factors other than DAmB dosage, and we are therefore unable to determine whether they account for the heterogeneity in mortality that is not explained by DAmB dosage.

The discordance between the influence that drug dosage has on CSF sterilization and mortality is reflective of a growing consensus that CSF sterility is just one of many determinants of mortality in cryptococcal meningitis. A systematic review of 27 clinical trials determined that there was no correlation between CSF sterility at 2 weeks and all-cause mortality at either 2 or 10 weeks (30). The most biologically plausible explanation for this is that fungal burden in the CSF may not reflect the extensive encephalitis that is characteristic of cryptococcal meningitis (which is more accurately termed “meningoencephalitis”). Histopathological defects are more marked in patients coinfected with HIV; fungi accumulate in perivascular spaces, are deposited [predominantly extracellularly] in brain parenchyma, and form granulomatous cryptococcomas in brain tissue (31, 32). It is conceivable that brain parenchymal damage is a dominant determinant of mortality and that clearance of fungi in CSF is not mirrored by clearance in the cerebrum and other CNS subcompartments. CSF sterility is an imperfect surrogate for the extent to which drug has penetrated into and sterilized the central nervous system.

The meta-analysis suggests a strong dose exposure-response relationship. Higher dosages are likely to be required to achieve efficacious drug exposure at the site of infection. DAmB has high molecular mass (924 g/mol) and complex binding properties (33). It does not readily penetrate the intact blood-brain barrier. Its concentration in meninges and cryptococcomas has been technically difficult to quantify in any finer detail than in brain homogenates in preclinical models (34, 35). This challenge is compounded by a lack of clarity regarding the DAmB concentration required for therapeutic efficacy at the site of infection. Animal studies have produced estimates indicating that the cerebral concentrations of DAmB at which the suppression of growth is half-maximal are 0.02 mg/liter in mice and 0.154 mg/liter in rabbits (34). AmB exposure above the level required to optimize antifungal activity appears to contribute only to toxicity (34, 36). Our simulations suggested that the optimal plasma AUC value in humans lies somewhere between 10 and 15 mg · h/liter, though the information required to extrapolate this to cerebral DAmB concentrations is not currently available. The application of noninvasive, high-resolution technologies, including matrix-assisted laser desorption ionization–mass spectroscopy imaging (MALDI-MSI), is now possible and offers the exciting potential to elucidate the pharmacokinetic/pharmacodynamic (PK/PD) index associated with efficacy at the site of infection by enabling quantification of drug in specific cerebral sites, as has been demonstrated in murine models that used gatifloxacin (37), doxycycline (38), pretomanid (39), and rifampin (40).

It may be the case that the maximal antifungal effect of DAmB is achieved with a dose of approximately 0.7 mg/kg, or slightly higher, and that gains made above this dose in terms of CSF sterility are offset by losses in terms of excessive toxicity. This may explain why significant increases in the proportions of patients achieving CSF sterility are not mirrored by reductions in mortality. The present analysis is not sufficient to more precisely define the optimal dosage of DAmB. This is partly due to the lack of consensus regarding DAmB exposure targets. We are unable to propose exposure targets based on our data set, which does not include site-specific PK or detailed toxicodynamic data. In addition, the pharmacodynamic and clinical outcome data presented here were derived from patient cohorts that were distinct from the patients that provided samples for the PK analysis. Outcomes from DAmB monotherapy at dosages of 0.7 mg/kg q24h and 1.0 mg/kg q24h have not been directly compared in a randomized controlled trial. However, comparison of these dosages in combination with 5FC has been performed. Bicanic et al. demonstrated increased early fungicidal activity with DAmB at 1 mg/kg q24h versus 0.7 mg/kg q24h, both in combination with 5FC at 100 mg/kg/day in four divided dosages, but this was not reflected in reductions in mortality. A higher percentage of deaths was seen in the higher-dose DAmB arm at both 2 weeks (9% versus 3%) and 10 weeks (26% versus 21%), but the data were not statistically significant (*P* = 0.62 and 0.77 at 2 and 10 weeks, respectively) (2).

In summary, these analyses suggest that the optimal dosage of DAmB for the treatment of cryptococcal meningitis lies between 0.7 and 1.0 mg/kg q24h. The precise drug exposure target that optimizes clinical outcomes without producing significant toxicity remains to be defined. The extent of interindividual PK variability in DAmB is modest and unlikely to account for the consistently poor clinical outcomes from treatment of cryptococcal meningitis.

## MATERIALS AND METHODS

### Clinical pharmacokinetic studies.

Plasma samples were obtained from adults with HIV-associated cryptococcal meningitis. Patients were initially recruited from a multicenter randomized controlled trial of adjuvant treatment with dexamethasone in HIV-associated cryptococcal meningitis, reported elsewhere (*n* = 3; International Standard Registered Clinical Number 59144167) (17). Following the early cessation of that trial, they were recruited from a prospective descriptive study at the same sites (*n* = 39). Patients were recruited at 2 sites: The Hospital for Tropical Diseases in Ho Chi Minh City, Vietnam, and Masaka General Hospital, Uganda. The study protocols were approved by the relevant institutional review boards and regulatory authorities at each trial site and by the Oxford University Tropical Research Ethics Committee.

The protocol for the randomized controlled trial has been described previously (41). Briefly, patients had HIV infection, a syndrome consistent with cryptococcal meningitis, and laboratory evidence of cryptococcal infection. Patients who were pregnant, had renal failure, had gastrointestinal bleeding, had received more than 7 days of anticryptococcal antifungal therapy, were already taking corticosteroids, or required corticosteroid therapy for coexisting conditions were excluded. The inclusion and exclusion criteria for the prospective descriptive study were identical to those of the clinical trial. Patients received DAmB at 1 mg/kg of body weight once daily by intravenous infusion over 5 to 6 h, as well as 800 mg fluconazole per day. Two patients recruited during the clinical trial received dexamethasone according to the following regimen: 0.3 mg/kg/day intravenously (i.v.) for week 1 and 0.2 mg/kg/day i.v. for week 2 and then orally at 0.1 mg/kg/day for week 3, 3 mg/day for week 4, 2 mg/day for week 5, and 1 mg/day for week 6, followed by cessation of treatment. For the first five patients enrolled, blood samples were obtained immediately prior to intravenous DAmB infusion and then at h 1, 2, 4, 8, 12, 16, 20, and 24. The results for these patients informed a subsequent sampling strategy defined using optimal design theory such that patients were sampled predose and then at 1, 2, 4, 8, 12, and 24 h after initiation of the infusion. PK sampling occurred on treatment days 1 or 2 and 7. Whenever patients had lumbar punctures performed for other clinical indications such as raised intracranial pressure, paired plasma samples were collected for subsequent PK analysis. Therefore, additional sparse samples were taken for up to 17 days after initial dosing. Quantitative fungal counts were determined for each lumbar puncture, as described previously (15).

### Measurement of amphotericin B concentrations.

Amphotericin B concentrations in plasma were measured using high-performance liquid chromatography (HPLC) with a Shimadzu Prominence HPLC system (Shimadzu, Milton Keynes, United Kingdom). Amphotericin B was extracted by protein precipitation. A total of 300 μl of methanol that contained piroxicam (Sigma-Aldrich, Dorset, United Kingdom) at 2 mg/liter was added as an internal standard to 100 μl of matrix. Samples were vortex mixed for 5 s and then centrifuged at 13,000 × *g* for 3 min.

A 150-μl volume of supernatant was removed and placed in a 96-well plate, to which 50 μl of water was added. A 50-μl aliquot was injected into a Kinetex 5μ XB-C18 liquid chromatography column (Phenomenex, Macclesfield, United Kingdom). Chromatographic separation was achieved using a gradient under starting conditions of 75% mobile phase A/25% mobile phase B (with 0.1% formic acid–water as mobile phase A and 0.1% formic acid–acetonitrile as mobile phase B). Mobile phase B was increased to 80% over 5 min and then reduced to the starting conditions for 2 min of equilibration. Amphotericin B and the internal standard were detected using UV detection at wavelengths of 406 nm and 385 nm; they eluted after 4.1 and 4.6 min, respectively.

The standard curve for amphotericin B encompassed the concentration range of 0.05 to 8.0 mg/liter and was constructed using blank matrix. The limit of quantitation was 0.05 mg/liter. The coefficient of variation was <9.3% over the concentration range of 0.05 to 8 mg/liter. The intraday variation and interday variation were <7.9%.

### Population pharmacokinetic modeling.

A PK model was fitted to the data using the nonparametric adaptive grid (NPAG) algorithm of the program Pmetrics (42) version 1.5.0 for R statistical package 3.1.1. The data were weighted using the inverse of the estimated assay variance. Both two- and three-compartment models were tested, with zero-order intravenous input and first-order elimination from the central compartment. The two-compartment model took the following form:

(a) $$\frac{dX(1)}{dt}=R(1)-\left[\frac{\text{SCL}}{V}+K12\right]\times X(1)+K21\times X(2)$$

(b) $$\frac{dX(2)}{dt}=K12\times X(1)-K21\times X(2)$$

(c) $$Y(1)=\frac{X(1)}{V}$$

where equations a and b describe the rates of change of the amount of drug (in milligrams) in the central and peripheral compartments, respectively. *X*(1) and *X*(2) represent the amounts of amphotericin B (in milligrams) in the central (c) and peripheral (p) compartments, respectively. *R*(1) represents the intravenous infusion of DAmB into the central compartment. SCL represents the first-order clearance of drug (in liters per hour) from the central compartment. *V* represents the volume of the central compartment. *K*12 and *K*21 represent the first-order intercompartmental rate constants. Equation c represents the model output. The three-compartment model contained the following additional equation to connect the third compartment to the second compartment in series:

(d) $$\frac{dX(3)}{dt}=K23\times X(2)-K32\times X(3)$$

An initial condition was estimated to accommodate detectable drug in the first PK sample from those patients who had received a dose of DAmB at an undocumented time before study enrollment. The nonzero initial condition was estimated by assigning the respective parameters in the structural model (not shown in the equations above). A switch was coded whereby a parameterized estimate of the initial condition was multiplied by a binary covariate equal to 1 (where the first PK sample was drawn after a dose of DAmB) or 0 (where this represented a predose sample).

Once the standard model (model 1) was fitted, the effects of patient weight, baseline eGFR, and patient ethnicity on the PK of DAmB were investigated. Bidirectional stepwise multivariate linear regression of each subject's covariates versus the Bayesian posterior parameter values revealed a significant (*P* < 0.05) relationship between both weight and eGFR and estimated PK parameters. Univariate linear regression was employed first to assess the relationship between patient weight and the Bayesian estimates for both clearance and volume. Since a positive relationship was observed between weight and both PK parameters, the population PK model was refitted to the data (model 2) with incorporation of the following equations to describe clearance (SCL) (equation e) and volume (*V*) (equation f) as functions of patient weight (Wt):

(e) SCL=Int _c+(Wt×Sl_c)

(f) V=Int _v+(Wt×Sl_v)

where Int is the intercept and Sl the slope of the linear regression describing the relationship between weight and clearance or volume; the intercept and slope for each of these PK parameters are parameterized separately. Thus, equation a of the structural model was replaced with the following equation:

(a.2) $$\frac{dX(1)}{dt}=R(1)-\left[\frac{{\text{Int}}_{c}+(\text{Wt}\times {\text{Sl}}_{c})}{{\text{Int}}_{v}+(\text{Wt}\times {\text{Sl}}_{v})}+K12\right]\times X(1)+K21\times X(2)$$

In addition, a power function was explored to describe the relationship between weight and clearance. In this model (model 3), clearance was parameterized and scaled with weight to the exponent 0.75. This exponent has previously been demonstrated to usefully scale for size (43, 44). A linear relationship was maintained between volume and weight. Thus, in model 3, equation a was replaced with the following equation:

(a.3) $$\frac{dX(1)}{dt}=R(1)-\left[\frac{\text{SCL}\times {\text{Wt}}^{0.75}}{V\times \text{Wt}}+K12\right]\times X(1)+K21\times X(2)$$

Univariate linear regression was similarly employed to assess the relationship between eGFR and the Bayesian estimates for clearance and volume from model 1. A weaker but nevertheless positive association was demonstrated. Consequently, a further structural model (model 4) was fitted to the data, with the following equation (equation g) employed to describe clearance (SCL):

(g) $$\text{SCL}=\text{I}\mathrm{nt}\_c+(\text{Wt}\times \text{Sl}\_c)\times \left(\frac{\text{eGFR}}{{\text{med}}_{\text{eGFR}}}\right)$$

where eGFR represents the estimated glomerular filtration rate calculated for each patient by the use of the Cockcroft-Gault equation and med_eGFR represents the population median estimated glomerular filtration rate. In model 4, equation a was replaced with the following equation:

(a.4) $$\frac{dX(1)}{dt}=R(1)-\left[\frac{{\text{Int}}_{c}+(\text{Wt}\times {\text{Sl}}_{c})\times (\frac{\text{eGFR}}{{\text{med}}_{\text{eGFR}}})}{{\text{Int}}_{v}+(\text{Wt}\times {\text{Sl}}_{v})}+K12\right]\times X(1)+K21\times X(2)$$

To explore whether there were significant differences between the model-predicted PK parameters in Vietnamese and Ugandan patients, Bayesian estimates of volume of distribution and clearance from the central compartment were compared using a Mann-Whitney test and Student's *t* test, respectively. Since no significant relationship between ethnicity and DAmB PK was apparent, this variable was not incorporated in the final model.

The fit of the model to the data was assessed using a linear regression of observed-versus-predicted values before and after the Bayesian step. The coefficient of determination of the linear regression was noted in combination with the intercept and slope of the regression for each model. Model comparison was achieved through calculation of the log likelihood value, the Akaike information criterion (AIC), the mean weighted error (a measure of bias), and the bias-adjusted, mean weighted squared error (a measure of precision). To verify the ability of the final model to predict observed concentrations with acceptable accuracy, a visual predictive check (VPC) of the data was performed. For the VPC, the covariance matrix in Pmetrics was utilized to simulate 1,000 patients administered DAmB on a milligram-per-kilogram basis. Simulated weight values were limited to the range observed in our clinical cohort.

### Meta-analysis of clinical outcome data.

The pharmacodynamic data from patients enrolled in the present clinical study are confounded by the coadministration of fluconazole (17). Therefore, a search was performed for clinical trials of treatment for cryptococcal meningitis with at least one arm comprised of adult patients receiving DAmB monotherapy. For consistency, the included trials were limited to those that recruited HIV-positive patients. Baseline clinical variables with a demonstrated ability to predict patient mortality—namely, altered mental status, patient age, and baseline CSF fungal burden—were selected *a priori* and extracted from the studies (4, 30). To aid meaningful trial comparison, baseline fungal burden and baseline CSF cryptococcal antigen titer values were extrapolated from one another where they were not explicitly reported in the study, applying a correlation presented by Jarvis et al. (4).

We collated a variety of clinical trial outcomes based on those that were commonly reported across trials of DAmB monotherapy: documented CSF sterility during trial follow-up, mortality at 2 weeks, and, where possible, mortality at 10 weeks. Meta-analysis was performed on each outcome using a dose-adjusted random-effects model to account for the baseline heterogeneity in the included studies. We included dose as a moderator variable in the model to assess the degree to which it explained heterogeneity in clinical outcomes (45). The resulting mixed-effects model took the form θ_*i*_ = β_0_ + β_1_dose_*i*_ + *u_i_*, where β_0_ and β_1_ are the model parameters intercept and dose, respectively; dose_*i*_ is the dose given in the *i*th study, assuming study-specific random effects; and *u_i_* = ∼*N*(0, τ^2^), where τ^2^ is the amount of residual heterogeneity among the true effects θ_*i*_ that is not accounted for by dose. We calculated to what extent dose as a moderator influenced the true average effect and estimated the corresponding proportions of each outcome measure.

### Monte Carlo simulation.

Monte Carlo simulations were performed in Pmetrics (42). Model 2 was used. Amphotericin B was administered on a milligram-per-kilogram basis and infused over 5.5 h. The initial conditions of the central and peripheral compartments were set at a default value of zero. The weight-based dosage of DAmB was converted to an absolute dosage by multiplying by the simulated patient's weight. This process served to mimic the bedside drug administration in the original clinical trial, in which dosing was planned on a milligram-per-kilogram basis but the absolute dose that was ultimately administered was determined by the patient's weight.

Drug exposure was quantified using the DAmB AUC (9, 10, 46). The simulated AUC for each patient was estimated 144 to 168 h post-therapy initiation. Simulations were performed to estimate the AUC that resulted from dosages administered in clinical trials of DAmB monotherapy for which PD measures were available—specifically, 0.4, 0.7, and 1.0 mg/kg q24h (5, 11, 13–15).
